# How do general practitioners handle complexities? A team ethnographic study in Japan

**DOI:** 10.1186/s12875-022-01741-8

**Published:** 2022-05-27

**Authors:** Junji Haruta, Ryohei Goto, Ozone Sachiko, Shuhei Kimura, Junko Teruyama, Yusuke Hama, Tetsuhiro Maeno

**Affiliations:** 1 Medical Education Center, School of Medicine, Keio University, 35 Shinanomachi, Shinjuku ku, Tokyo, 160-8582 Japan; 2 Department of Primary Care and Medical Education, Faculty of Medicine, University of Tsukuba, Tsukuba, Japan; 3 Faculty of Humanities and Social Sciences, University of Tsukuba, Tsukuba, Japan; 4 Faculty of Library, Information and Media Science, University of Tsukuba, Tsukuba, Japan; 5 Tokyo Junior College of Transportation, Tokyo, Japan

**Keywords:** Complex adaptive system, General practitioners, Team ethnography

## Abstract

**Background:**

General practitioners (GPs) are often faced with complex problems, including patients with socio-economic and medical problems. However, the methods they use to approach these complexities are still not understood. We speculated that elucidating these methods using complex adaptive systems (CAS) methodology to comprehensively assess GPs’ daily activities would contribute to improving the professional development of GPs. This study aimed to clarify how expert GPs handle complex problems and adapt to their community context through the ethnography of GPs and other healthcare professionals in terms of CAS.

**Methods:**

We adopted the interdisciplinary team-ethnographic research approach. Five hospitals and four clinics in Japan which were considered to employ expert GPs were selected by purposive sampling. 62 individuals of various backgrounds working in these nine facilities were interviewed. Using field notes and interview data, the researchers iteratively discussed the adequacy of our interpretations. The first author (JH) prepared a draft report, which was reviewed by the GPs at the participating facilities. Through critical and iterative consideration of the different insights obtained, the final findings emerged together with representative data.

**Results:**

We identified four approaches used by GPs to deal with complexities. First, GPs treat patients with complex problems as a whole being and address their problems multi-directionally. Second, GPs build horizontal, trusting relationships with other healthcare professionals and stakeholders, and thereby reduce the degree of complexity of problems. Third, GPs change the learning climate while committing to their own growth based on societal needs and by acting as role models for other professionals through daily interpersonal facilitation. Fourth, GPs share community vision with multi-professionals and thereby act as a driving force for organizational change. These various interactions among GPs, healthcare professionals, organizations and communities resulted in systematization of the healthcare and welfare network in their community.

**Conclusions:**

Expert GPs developed interconnected multidimensional systems in their community health and welfare networks to adapt to fluctuating social realities using four approaches. GPs’ work environment may be considered as a complex adaptive system (CAS) and the approach of GPs to complexities is CAS-based. Our findings are expected to have practical applications for GPs.

## Background

Healthcare professionals in developed countries are frequently puzzled and confounded by complex problems, such as the increase in comorbidities and inequality. In particular, general practitioners (GPs) commonly examine patients with multiple complex conditions, defined by the Agency for Healthcare Research and Quality as those with two or more chronic conditions which may collectively have an adverse effect on health status, function, or quality of life, and that require complex approaches to healthcare [1].

GPs are experts at dealing with newly complex models which incorporate mental health and social effects, as well as with factors which exacerbate complexity, such as medical disorders, psychiatric disorders, socioeconomic problems, behaviors and characteristics, or their combination [2–5]. GPs provided appropriate advice to patients who attend multiple specialties and do not know whom they should consult [6]. Additionally, GPs try to identify conditions which are both suitable for their communities and which meet the community’s expectations and satisfy its values [7].

Despite this experience, GPs find that clarifying their role in the handling of complex problems is difficult. This difficulty owes to the need to grasp these problems simultaneously in the context of individual patients and communities [6]. Moreover, the approaches to complex problems that expert GPs use to adapt to their community context are unclear. Complexity science and complex adaptive systems (CAS) could be useful in clarifying how complex problems are coped with, since these perspectives represent an alternative to the reductionist view [8]. Complexity science, which is rooted in both nonlinear mathematics and coordination dynamics, focuses on relationships among variables and allows for emergent behaviors [9]. In contrast, CAS is a network-based system which includes agents whose actions are interconnected within the system according to patterned behavior. To clarify these patterned interactions in CAS, which are defined by the presence of regular sets of coordinated behavior (i.e., verbalizations and nonverbal actions), repeated over time and occurring above and beyond chance [10], the whole system needs to be observed functioning within its context. We therefore speculated that ethnography,[11] which explores cultural phenomena from the perspective of the subject, may allow an unravelling of the many multi-faceted and multilayered viewpoints GPs use to clarify these approaches. Further, we considered that elucidating them in terms of CAS would contribute to research into GPs’ professional development, and inform educational practices aimed at supporting this research.

Of note, the Japanese healthcare system does not have a patient list system or registration system. In Japan, general practice does not function in a strict gatekeeping role: people can access secondary and tertiary care facilities directly without a referral from a primary care physician. In addition, the boundaries between primary/secondary care and clinic/hospital care are unclear, and patients can freely choose and change their physician or healthcare facility without a referral from a GP. As a result, 7 million of Japan’s 127 million population are estimated to visit specialists as outpatients, with hospitals accounting for 30% of these visits [12]. Nevertheless, to promote the continuity of care between inpatient and outpatient status, the Japanese government has incentivized the holding of an interprofessional conference before a patient is discharged from hospital to a community home. In other words, physicians in hospitals need to provide not only secondary but also primary care, while GPs mainly provide primary care in the general practice departments of hospitals and small outpatient clinics [13]. It is within this ill-defined context that expert GPs in Japan are struggling with complex problems.

Here, we aimed to clarify how expert GPs handle complex problems and adapt to their community context through the ethnography of GPs and other healthcare professionals in terms of CAS in Japan.

## Methods

### Design

We undertook qualitative research based on the team ethnography methodology. To clarify the role of GPs as agents in CAS, which is focused more on the relations and interconnection among individuals rather than their characteristics as individuals, we adopted the ethnography method, as “occurring in natural settings characterized by learning about the culture of the group under study and experiencing their way of life before attempting to derive explanations for their attitudes and behaviors” [14]. Using the interdisciplinary team ethnography method, we arranged for a pair of researchers to engage in participant observation and interview together, in the same place and at the same time [11]. The team consisted of a healthcare professional(s) and/or an anthropologist, who conducted fieldwork in a medical facility together.

### Research setting

The research setting was Japanese hospitals or clinical settings with expert GPs. The Japan Primary Care Association began certifying primary care specialists in 2010, and had certified 874 specialists as of September 5, 2020 [15].

Regarding complex problems, a survey of Japanese people aged 75 and older living in Tokyo found that about 65% or more had three or more comorbidities, with the most common three-way pattern being hypertension, coronary heart disease, and peptic ulcer disease in men, and hypertension, dyslipidemia, and peptic ulcer disease in women, in that order [16]. Identified patterns of multimorbidity in the Japanese general population were cardiovascular-renal-metabolic, neurological-psychiatric, skeletal-articular-digestive, respiratory-dermatologic, and malignancy-digestive-urologic [17]. Among home visit patients, 50% have multimorbidities; cardiovascular, endocrine, and neuropsychological disorders are common, and are associated with depressive symptoms, indicating the importance of the collection and appropriate utilization of psychosocial information [18]. With the increase in lifestyle and other non-communicable diseases, these complex problems involving multiple biopsychosocial factors are an increasingly more important priority for GPs. Although the number of studies on multimorbidity has recently increased, we are unaware of any studies evaluating multimorbidity from a CAS standpoint. In addition, it is suggested that physicians themselves may feel a degree of burden concerning complex problems due to the lack of a view of CAS [19, 20]. We therefore hypothesized that Japanese GPs may not fully understand the concept. In this study, therefore, we purposefully sought out GPs who might be experts in hospitals and clinics to reveal multiple aspects of their behavior as agents in CAS.

### Participants

Purposive sampling was adopted. Given the research setting, sampling was conducted with comprehensive consideration to the criteria used to characterize expert GPs and facilities, existing relationships within the local region, size of the municipal population, presence of public medical institutions, and the scale and history of the facilities.

First, we selected GPs who had been working full time for more than 5 years (10,000-h expert theory [21] in their context. In addition, we purposefully selected three types of expert GPs identified in a previous paper [22] and the facilities they belong to. The first type were all-rounder GPs who could deliver specialist-defined care in a wide range of areas. All-rounder GPs, who have a wide range of specialist knowledge as well as the ability to work on inter-professional and inter-departmental relationships, often work in the general practice department of hospitals. The second type were GPs with appropriate experience who were able to independently deliver a specialist service [23]. GPs with special interests, such as gastrointestinal endoscopy, often work in rural hospitals since more physicians work in urban areas than rural areas. The third type were expert generalists who could interpret patient problems in their local community, and who mainly work in clinics. Additionally, considering a Japanese study by the Health Labor Sciences Research Grant System in 2018 on the effect of GPs on the community [24], we examined five hospitals and four clinics as model facilities in which expert GPs practice. This paper does not describe the specific facilities associated with the following findings to avoid the identification of participating facilities and individuals.

### Data collection

The researcher conducted pilot studies in two facilities in Japan, and based on these developed a semi-structured interview guide (Table 1).

**Table 1:** Semi-structured guide for interviews with GPs and healthcare professionals

For GPs in their institution	For healthcare professionals
What types of clinical tasks have you performed as a GP in your institution?	What types of clinical tasks have you performed in your institution with GPs?
What do other healthcare professionals think about your tasks and how you work in the organization?	What do you think about the GPs’ tasks in your institution and how you work with them in the organization?
How do you work and collaborate with other professionals and/or other departments in your organization/local community?	How do the GPs in your institution work and collaborate with other professionals and/or other departments (including you) in your organization/local community?
What kinds of collaboration do you have with neighboring organizations?	What kinds of collaboration do you have with neighboring organizations?
How and which professionals are mainly responsible for internal and external collaboration in each organization? How have GPs (including you) been involved in this process?	How and which professionals are mainly responsible for internal and external collaboration in each organization? How have GPs been involved in this process?
How has your organizational culture developed, and who has been involved? How have the GPs in your institution been involved in this process?	How has your organizational culture developed, and who has been involved? How have GPs in your institution been involved in this process?
How has your organization approached patients and lay people in the local community? How do you think they feel about it? What impact do you think this approach has had on patients and community members? How have the GPs (including you) been involved in this process?	How has your organization approached patients and lay people in the local community? How do you think they feel about it? What impact do you think this approach has had on patients and community members? How have GPs in your institution been involved in this process?

The study included nine medical institutions located across Japan. After participants and their facilities provided e-mail consent to participate, our research team conducted participatory observation at each facility in February, August, and November 2019 and February 2020 (Table 2).

**Table 2:** Participant facilities, interviewed subjects, and combinations of researchers

	Expert GPs Age (gender)	Region and municipality	Population composition of the municipality [As of January 2020.]	Administration	Scale of the hospital or clinic	History of the general practice facility	Interviewed subjects	Schedule of visit (period)	Researchers
Hospital A and an affiliated outpatient and home visit clinic	40 s (M^a^) and 40 s (M^a^)	Kansai, a local small city located in the suburbs	Approximately 80,000 people	Public	Regional core general hospital with 350 beds	General internal medicine department started in 2008	Five physicians (four GPs (M) and GP residents (F)), one physical therapist (M), two nurses (F), one MSW (F), and one medical assistant (F)	February 21, 2019 (1 day)	JH, JT
Clinic B	50 s (M^a^) and 30 s (M^a^)	Kansai, a rural town	Approximately 10,000 people	Private	Outpatient and home visit clinic (no hospitalization)	Clinic started in 1999	Two male GPs (due to limited time, physicians only)	February 21, 2019 (1 day)	SO, SK
Hospital C	50 s (M^a^)	Hokkaido, a central city of the prefecture	Approximately 2,000,000 people	Private	Regional core hospital with 500 beds	General practice department started in 2002	Three GPs (M) and an emergency medicine specialist (M), three nurses (F), one physical therapists (M), one MSW (M)	August 30–31, 2019 (2 days)	JH, SK
Clinic D	40 s (M^a^)	Hokkaido, the same city as Hospital C	Approximately 2,000,000 people	Private	Hospital-affiliated outpatient and home visit clinic	Clinic started in 2000	One GP (M), two nurses (F), one nutritionist (F), one MSW (F), one medical clerk (F), one administrative staff (M), one nurse (F) in a nearby care facility	August 29–30, 2019 (2 days)	SO, YH
Clinic E	40 s (M^a^)	Hokkaido, the same city as Hospital C	Approximately 2,000,000 people	Private	Outpatient and home visit clinic	Clinic started in 2010	One GP (M), one nurse (F), one physical therapist (M), one MSW (F), one chief of medical staff (F), one nurse (F) in a nearby care facility	August 29–30, 2019 (2 days)	RG, JT
Hospital F	40 s (M^a^)	Kanto, a central city of the prefecture	Approximately 500,000 people	Public	Regional core hospital with 350 beds	General practitioners have been engaged there since 2011	One GP (M), one nurse (F), one pharmacist (M), one physical therapist (M), two MSWs (F), one chief of medical staff (M)	November 21, 2019 (1 day)	JH, RG
Hospital G	50 s (M^a^)	Okinawa, a central city of the prefecture	Approximately 120,000 people	Public	Regional core hospital with 500 beds	General internal medicine center started in 1996 (discontinued during fiscal year 2006 to 2007, and resumed in fiscal year 2008)	Three GPs (M), one nurse (F), one medical staff (F)	December 19, 2019 (1 day)	JH, RG, JT
Clinic H	50 s (M^a^)	Tohoku, a central city of the prefecture	Approximately 230,000 people	Private	Clinic	Clinic started in 2010	One GP (M), one nurses (F), three medical clerks (F),	February 10–11, 2020 (2 days)	JH, RG
Hospital I	40 s (M^a^)	Kyushu, a city located in the suburbs	Approximately 130,000 people	Private	Hospital	Transferred from municipal to private status in 2008	Two GPs (M), three nurse (F), one pharmacist (F), one physical therapist (M), one speech therapist (F), one medical clerk (F), two MSWs (M, F), one medical secretary (F)	February 20–21, 2020 (2 days)	SO, SK, YH

^a^*M* Male, *F* Female

The researchers conducted participatory observations, including a tour of the facility with the GPs or GP-recommended staff members who were the key informants of the participating facility [25], and observed that GPs were engaged in outpatient treatment and case conferences, etc. The GP also introduced the team to key participants who agreed to be interviewed. The researchers interviewed individual participants including doctors, nurses, rehabilitation therapists, and medical social workers MSWs) for 30 min to one hour each in a private room without the presence of other facility colleagues (Table 2). In the case of interviews with multiple researchers, one researcher was the main interviewer and the other researcher acted as note-taker and sub-interviewer. All audio records of interviews were transcribed verbatim. Through participatory observations and interviews, the researchers collected data based on differing viewpoints (i.e. those of healthcare professionals, non-professional staff, local people, and others) on how GPs work in the specific context.

Initially, interdisciplinary team ethnography of two facilities (Hospital A and Clinic B) was conducted from the perspective of “Medical Generalism” as a principle in primary care [26]. Based on the results of this fieldwork, we determined that the CAS was a useful way to capture the practices of GPs as “Medical Generalism” by reflecting interactions among individuals-departments-organizations-communities as a means of understanding complexity [27]. Thus, the CAS was adopted as a framework for data collection and analysis to clarify the perspective of this study. This method is reflected in the structure of the findings.

The researchers interviewed 62 individual healthcare professionals in the participating medical institutions. Hospital A is a central public hospital in a town with a population of slightly less than 80,000 people in the Kansai district of Japan. Clinic B is a private clinic in a rural town of approximately 10,000 people neighboring that of Hospital A. Hospital C is in a large city in Hokkaido with a population of more than 1,000,000. Clinic D is affiliated with a hospital. Clinic E, located in the same city as Hospital C, was converted to a general practice clinic by a GP when the original orthopedic surgery clinic was closed. Hospital F is in the North Kanto district. Hospital G is on the main island of Okinawa. Clinic H is in the Tohoku area. Hospital I is a small hospital in the suburbs of Kyushu. The research team assumed that all-rounder GPs worked in Hospitals A, C, F and G; GPs with special interests such as HCU (High Care Unit) worked in Hospitals C and F; and GPs as expert generalist worked in the Hospital A-affiliated clinic, Clinics B, D, E, and H, and Hospital I, and selected GPs in these centers as participants.

### Researcher backgrounds

JH and SO are general practitioners and RG is a physical therapist. JH received training in qualitative research as part of a PhD program, while SO and RG received qualitative research training after obtaining their PhD degrees. SK is a cultural anthropologist and JT and YH are medical anthropologists, each with about 10 years’ experience. The authors have collaborated since January 2018.

### Analysis

Ethnography involves data collection and analysis. Each researcher composed his or her own field notes and shared them within the group. The researchers held multiple face-to-face meetings every two or three months. Each meeting was an opportunity to discuss iteratively the adequacy of our interpretations. Researchers constructed and deconstructed certain patterns in the way GPs handled complex problems and adapted to their community context through the sharing and comparing of their field notes. Through continued discussions with the anthropologists on the team, who stressed the significance of clinical and social science perspectives, however, we tried to be continuously reflexive during the analysis in order to practice more interpretive reflexivity. After the findings were finalized, the first author (JH) prepared a draft report. The GPs at the participating facilities reviewed this draft and were asked for their opinions on it. Through these processes, the final findings emerged together with representative data and were confirmed by all researchers. This study was reviewed and approved by the research ethics committee of the authors’ university.

## Results

In these Japanese primary care settings, all three types of expert GPs, namely all-rounder GPs, GPs with special interests, and expert generalists, were found to face more or less multifocal levels of complex problems in the context of individual patients, healthcare professionals, organizations, and communities. In the system, we focused on GPs’ interactions among individuals. Among examples, GPs shared information with other healthcare professionals about complex patients with multiple chronic conditions, etc. On the other hand, some organizations have trouble with sharing clinical information with healthcare professionals and management of healthcare organizations due to sectionalism, etc. Some communities have concerns about the optimal use of community resources and interprofessional collaboration strategies beyond their facilities. In such contexts, the final findings revealed that four types of patterned GP interaction between GPs and other healthcare professionals facilitated a reduction in these complexities throughout the network and in the necessary parts of the network, namely:

1. GPs shared the whole picture of patients with complex problems and address their problems multi-directionally.
2. GPs built horizontal, trusting relationships with other healthcare professionals and stakeholders to share information and thereby reduce the degree of complexity of problems.
3. GPs changed the learning climate while committing to their own growth based on societal needs by reflecting on their own position and working as role models for other professionals through daily interpersonal facilitation.
4. GPs shared community vision with multi-professionals as a driving force for organizational change.

1. GPs share the picture of patients with complex problems as a whole being and address their problems multi-directionally

GPs perceived the individual patient as a whole being and recognized the interrelationships of health risk factors with patient issues such as polypharmacy with multiple chronic conditions. GPs have willingly tried to incorporate multi-dimensional perspectives on the complexity, which enabled healthcare professionals to reconsider their roles.

*“I get the impression that GPs have a broad perspective (on their patients). The GPs have a broad view (of the patient), and also looks at the future and the background. Even a single record is different. In the emergency room, they tend to just describe the symptoms, but they also describe where the patient is coming from and his or her family.” (nurse in Hospital C)* *The specialist said, “It takes multiple perspectives on the patient, which makes subsequent diagnosis and treatment easier. It would have been very difficult without them. (fieldnote in Hospital C).*

Additionally, by involving other healthcare professionals and being involved, GPs reduced not only the complexity of multimorbidity care, but also social, economic, and mental difficulties by internalizing the bio-psycho-social approach.

*“Administration staff sometimes accept patients with intractable and multiple diseases to our hospital. In these cases, we introduce GPs to them.” (nurse in Hospital C)*

*“I think GPs always talk with patients about their economic and physical conditions.” (MSWs in Hospitals A, C and F)*

Similar thoughts were expressed by nurses and social workers, as follows: *“To be honest, we are specialists who support patients’ QOL and post-discharge lives rather than the disease, but the GPs are equally concerned about the psychosocial issues of their patients, so that's really, in a good way, something we shouldn't lose. “(field note in hospital F).*

Thus, GPs shared in the care of individual patients as a whole being and addressed their problems multi-directionally through multi-dimensional perspectives with multi-professionals. As a result, healthcare professionals improved their understanding of the prioritization of disseminated issues and were able to advance their provision of care in a stable manner. Through GPs’ interactions, the sharing of the picture of patients with complex problems triggered interconnections with the other healthcare professionals’ view as part of the network.

2 GPs build a horizontal, trusting relationship with other healthcare professionals and stakeholders and thereby reduce the degree of complexity of problems.

GPs helped multi-healthcare professionals understand complex information while working with a sense of reciprocity, which means that they helped and complemented the activities of other healthcare professionals voluntarily and horizontally, instead of in a hierarchical, top-down relationship with them.

*“Now, each GP has a conference once a week with physicians in charge, nurses, MSWs, nutritionists, therapists, and pharmacists to discuss whether any current patients can be discharged. This is a very beneficial conference for healthcare professionals in our department as it allows them to understand medical information in summary form.” (nurses in Hospital C)*

*“We are frankly working with the GPs to come up with solutions to the complex information surrounding our patients. If this is the only conclusion we can reach now, we can agree. We will then feel reassured, and be able to say, “Well, we'll just have to wait and see.” (pharmacist in Hospital F)*

The GPs carefully exchanged complicated internal and external information regarding their own departments and organizations, and reduced the degree of complexity of information to meet the activities of their departments, organization and community.

*“Working with GPs has opened my eyes to the outside world as well. We have many opportunities to exchange information about management systems at other facilities with GPs”. (nurse in Hospital I)*

*“We established a relationship with a community newspaper company to deliver meaningful medical information to lay people. We then started publishing easy-to-understand information on important subjects in family medicine for lay people, which was very well understood by patients. For example, I wrote about advanced care planning. After that, some patients started to tell me that they wanted to reflect on their own end of life. Recently, the company has increased the number of opportunities for us to publish articles about medical information”. (GP in Clinic H)*

Thus, the findings revealed that these patterned interactions between GPs and other healthcare professionals and/or community stakeholders established horizontal and trusting relationships which were interconnected with a reduction in the degree of complexity of information to meet particular contexts as part of the network.

3 GPs change the learning climate while committing to their own growth based on societal needs through reflecting on their own positions and acting as role models for other professionals through daily interpersonal facilitation.

GPs reflected on their own position in the organization and local community, and expanded their own duties or created innovative work behaviors based on societal needs.

*“We had not previously established a home care system. We therefore developed home visiting nursing-care and rehabilitation programs, because such systems were necessary in our local community. We achieved interprofessional collaboration through the development of these systems.” (GP in Clinic B)*

*The GPs took time to gain an understanding of common background matters and to strengthen human networks with care managers and other professionals under the concept of the “community hospital,” which provides comprehensive and continuous care in the community. (Fieldnote in Hospital I)*

Additionally, GPs acted as role models and aided the other healthcare professionals in reflecting on their work, which tended to be focused around procedures and efficiency of care.

*“We have been learning about how to collect the family history and background of patients - which is part of our responsibilities - through study meetings with our GPs.” (nurse at an HCU in Hospital C).*

*In evening conferences, a GP was asking questions in an attempt to broaden healthcare professionals’ perspectives and made statements that caused the healthcare professionals to reflect. Another GP allowed the healthcare professionals to speak freely. GPs, by adopting different patterns of respectful attitude - as appropriate for the character of the particular healthcare professional they were dealing with - used bottom-up communication strategies so that all individuals could think about the patient’s ‘best life’. (fieldnote at Clinic D)*

In this way, the GPs gradually changed the learning climate in the department.

Thus, GPs undertook both self-improvement and improvement of other healthcare professionals through interpersonal facilitation based on the local context. The patterned interactions between GPs and other healthcare professionals and/or organizations empowered them to commit to their growth. Through this process, the individuals reflected on their own positions and were motivated to learn what was necessary to care for their patients.

4 GPs shared community vision with multi-professionals as a driving force for organizational change

GPs routinely came across opportunities to remind other healthcare professionals and staff of their goals, and stimulated their morale and motivation.

*GPs held routine meetings with healthcare professionals every morning and once a week in the evening, which had the effect of increasing staff morale. (fieldnote at Clinic C)*

*“We conduct daily visiting rounds with our GPs, which facilitates patient transfer or discharge from hospital. Although it is not easy to achieve our goal, we learn a lot from the GPs’ viewpoint during these rounds.” (nurse working in the local community network section of Hospital C, who lead the rounds in the ward)*

*Similar data were extracted from Hospitals A* *,* *F* *,* *G, and I.*

GPs also played the role of authority, leader, and collaborating motivator in efforts to change the system of their organization or institution.

*When they contacted other facilities to transfer their own patients, they noted, “We, as team members, carry out our mission by getting advice from GPs. Hospitalization systems have improved year by year because we changed our system to meet requests from other medical institutions while receiving advice and authorization from GPs.” (fieldnote at Hospital C)*

*Similar data were extracted from Hospitals A* *,* *F* *,* *G, and I.*

In this way, GPs emphasized community values as a means of inspiring healthcare professionals and staff to reflect on their behavior in the service of patients, facilities, and their local community and to achieve sustainable local community development. At the same time, they accepted the efforts of others, shared their goals, and worked together with them to achieve the goals. GPs served as role models for healthcare professionals. As such, from the patterned interactions between GPs and/or other healthcare professionals and/or organization and/or community emerged a shared vision as a driving force for organizational change. These interrelations systematized their healthcare and welfare community networks.

## Discussion

This study showed that GPs try both to make sense of complex problems as whole networks and to construct personal relationships using four types of approach: (1) realize the complex problems of individual patients as whole beings from a multi-dimensional perspective; (2) build a horizontal, trusting relationship with stakeholders by reducing the degree of complex information; (3) change the learning climate while committing to their own growth based on societal needs through reflecting on their own position and acting as role models for other professionals through daily interpersonal facilitation; and (4) sharing a community vision as a driving force for organizational change.

The core role of GPs, namely to deal with multiple comorbidities in a comprehensive manner, requires that they treat patients with the awareness that biopsychological factors and social networks that arise from the combination of chronic diseases modify health outcomes and quality of life [28]. The European Society of General Practice/Family Medicine has also called for the provision of comprehensive patient-centered care and quality improvement for such patients [29]. These behaviors, including interactions among individuals-departments-organizations-communities, can be considered indicative of a complex adaptive system (CAS) [30]. The first two approaches are consistent with the classical CAS concept from a historical perspective. [31] Classical CAS is understood as one of the frameworks associated with patients in health, disease, and illness, and as a multivariable, non-linear, and nonperiodic system for understanding context-dependent individuals, departments, organizations, and communities [32–34]. When transformational leaders consider organizational structures as complexity systems, their organizations are able to transform into well-functioning practices [34]. In addition, GPs are required to cocreate organization dynamics in order to capture multidimensional properties at multiple levels and adapt to internal and external changes, which is also the case when patient well-being is promoted [35].

Moreover, the last two types of approach showed that GPs themselves acted as a learning organization, identified team functions which are linked to an interprofessional competency framework [36], and made dynamic changes which appear to arise in a bottom-up manner rather than as a hierarchical structure [37]. GPs might therefore enhance morale by showing community values through stimulation of reflection by healthcare professionals and staff, and also play a leading role in organizational direction. They achieve both of these goals by showing a high level of interest in multi-stakeholders, combining the dynamics of multilevel and multidimensional changes, and approving and motivating the staff involved. Some papers have investigated these interconnected multidimensional systems as an exploratory theory in the primary care area [38–41].

The findings revealed that the patterned interactions of GPs and patients, healthcare professionals, organizations, and communities transformed their roles in the context of the whole network. These interconnections created the trustworthy relationships between GPs and healthcare professionals. Such relationships fostered a psychologically safe environment within which they could take risk-taking action [42]. Once such psychological safety was in place, GPs committed themselves to self-improvement in response to the changing organizations and community, and the interprofessional team were in turn empowered by these changes. When a shared community vision was clarified from these interactions, individual agendas could be systematized. Not only hospitals and clinics, but also whole organizations and communities could be organized in a little over a year by the emergence of expert GPs. Thus, expert GPs played a central role in the development of interconnected multidimensional systems within the community networks of health and welfare aimed at adapting to fluctuating social realities.

### Strengths and limitations

Interdisciplinary team ethnography is a relatively effective research design because large amounts of data can be collected at the same time by relatively few researchers. Conducting visits by a medical professional and an anthropologist as a pair was helpful in sharing the healthcare context and in identifying interpretations and perspectives from different angles.

However, funding issues limited data collection to one or two days of field work at each facility. In addition, we should emphasize that not all of the changes described above were observed in all facilities. In addition, since the findings are based on the analysis of data obtained from interviews with GPs and other healthcare professionals and observations of facilities, it may not capture the entirety of CAS, which is focused on the interconnections of the system components, such as potential rules and individual behaviors in patient care. Pragmatic change takes longer in some organizations, especially hospitals, because they have many stakeholders. Additionally, CAS, in which changes in one element can mutually alter the whole, may be difficult for GPs and health professionals to understand. Not all of the networks were smoothly established, including relationships with GPs, healthcare professionals, specialists, lay people in the community, and the government. However, as patterns of CAS in primary care practice emerge, it is meaningful that expert GPs can exert different outcomes on contextual change using these four CAS-based approaches when they collaborate with key persons in the context of healthcare professionals, organizations and communities. As part of an overall goal to share a whole and complete picture of dealing with complex problems in the primary care field, our identification of four types of specific CAS-based approach in this study is meaningful not only for GPs but also for other healthcare professionals who are also required to cope with complexity.

Considering the GP system and culture in Japan, which has a relatively limited history and is still developing [14], the patterns of interaction of GPs with other healthcare professionals emerged, identifying them as complex adaptive agents of the healthcare organization in responding to the complex problems of patients, families and communities that can be affected by social change. These patterns were namely identified as four specific CAS-based approaches to complex problems. Increasingly complex problems require GPs to be the driving force behind organizational growth to expand the diversity of primary care service delivery. This is expected to have practical applications for GPs.

This expectation is strengthened in view of the relative paucity of other papers on this topic. Further, these findings will be useful in helping GPs in other countries to express their roles in complex systems.

## Conclusion

GPs create interconnected multidimensional systems while trying to both make sense of complex problems as a whole network and construct interpersonal relationships through four CAS-based approaches.

## Data Availability

The interview data and field note collected and analyzed during the current study are not publicly available because we did not receive informed consent concerning data sharing from the participants.

## Abbreviations

GP: General practitioners
CAS: Complex adaptive systems
